# Supplementary material for: Changes in Physical Fitness Parameters in a Portuguese Sample of Adolescents during the COVID-19 Pandemic: A One-Year Longitudinal Study
Source: Int J Environ Res Public Health. 2023 Feb 15;20(4):3422. doi: 10.3390/ijerph20043422 (PMC9965852; doi:10.3390/ijerph20043422)
Supplement: Supplementary file 1 [file ijerph-20-03422-s001.zip › ijerph-2160546-supplementary.pdf]

Table S1. Mixed model ANOVA and Bonferroni Pairwise Comparisons of sex differences in Early adolescents.

		Pre-Pandemic	Pandemic				Bonferroni Pairwise Comparisons		
		**T1 (Dec 2019)** *M* *(SD)*	**T2 (Oct 2020)** *M* *(SD)*	**T3 (Dec 2020)** *M* *(SD)*		**η2**	**T1** *vs.* **T2**	**T2** *vs.* **T3**	**T1** *vs.* **T3**
**Body Composition**									
BMI HFZc (kg/m^2) (*n* = 421)	**Girls** (*n* = 198)	.22 (19.65)	2.05 (20.79)	1.06 (20.10)	*F* (1.373, 575.267) = .055 (*ns*)	.000	*ns*	*ns*	*ns*
	**Boys** (*n* = 223)	4.73 (23.09)	6.22 (22.03)	5.53 (21.14)			*ns*	*ns*	*ns*
WC (cm) (*n* = 90)	**Girls** (*n* = 45)	78.42 (11.37)	79.12 (10.26)	77.92 (10.69)	*F* (1.874, 164.946) = .985 (*ns*)	.011	*ns*	*ns*	*ns*
	**Boys** (*n* = 45)	80.70 (11.29)	82.89 (10.20)	81.87 (9.24)			*ns*	*ns*	*ns*
**Aerobic Fitness**									
VO_2max (ml/kg/min)	**Girls** (*n* = 193)	40.40 (3.18)	38.87 (3.08)	39.37 (3.28)	*F* (1.685, 697.545) = .871 (*ns*)	.002	***	*	***

(*n* = 416)	**Boys**	43.57	41.78	42.66			***	***	***
	(*n* = 223)	(5.78)	(5.11)	(5.53)					
**Neuromuscular Fitness**									
	**Girls**	21.54	22.88	25.44			*ns*	*ns*	*
Sit-ups	(*n* = 140)	(15.65)	(16.09)	(17.70)	*F* (1.772, 581.130) = .970 (*ns*)	.003			
(*n* = 330)	**Boys**	26.25	29.70	32.42			*	*	***
	(*n* = 190)	(17.62)	(19.33)	(20.78)					
	**Girls**	7.11	6.98	7.43			*ns*	*ns*	*ns*
Push-ups	(*n* = 155)	(6.05)	(5.37)	(5.71)	*F* (1.603, 548.330) = .585 (*ns*)	.002			
(*n* = 344)	**Boys**	10.79	11.02	11.81			*ns*	*	*ns*
	(*n* = 189)	(8.03)	(6.83)	(7.35)					
	**Girls**	120.99	127.65	129.41			***	*ns*	***
Horizontal Jump	(*n* = 157)	(22.14)	(23.97)	(24.51)	*F* (1.882, 617.393) = 8.927***	.026			
(*n* = 330)	**Boys**	136.17	149.00	152.19			***	*	***
	(*n* = 173)	(26.76)	(30.37)	(30.79)					
	**Girls**	4.53	4.59	4.59			*ns*	*ns*	*ns*
20m Run	(*n* = 173)	(.54)	(.53)	(.55)	*F* (1.540, 574.400) = 6.488**	.017			
(*n* = 375)	**Boys**	4.32	4.23	4.22			*	*ns*	*
	(*n* = 202)	(.62)	(.51)	(.52)					

	**Girls**	19.39	23.36	23.42			***	*ns*	***
Sit and Reach	(*n* = 158)	(7.39)	(7.74)	(7.75)	*F* (1.556, 541.327) = 8.067**	.023			
(*n* = 350)	**Boys**	15.50	17.82	17.40			***	*ns*	***
	(*n* = 192)	(6.62)	(7.27)	(7.02)					

*p < .05, **p < .01, ***p < .001; *ns* – non-significant value; BMI HFZc – body mass index health fitness zone continuum; WC – waist circumference; VO2max - maximal oxygen uptake;

Table S2. Mixed model ANOVA and Bonferroni Pairwise Comparisons of sex differences in Middle adolescents.

		Pre-Pandemic	Pandemic				Bonferroni Pairwise Comparisons		
		**T1 (Dec 2019) *M* (*SD*)**	**T2 (Oct 2020) *M* (*SD*)**	**T3 (Dec 2020) *M* (*SD*)**		**η_p2**	**T1 *vs.* T2**	**T2 *vs.* T3**	**T1 *vs.* T3**
**Body Composition**									
	**Girls**	-2.95	-3.72	-3.72			*ns*	*ns*	*ns*
BMI HFZc (%)	(*n* = 44)	(18.37)	(19.10)	(19.37)	*F* (1.423, 136.630) = .452 (*ns*)	.005			
(*n* = 98)	**Boys**	-6.96	-6.62	-7.58			*ns*	*ns*	*ns*
	(*n* = 54**)**	21.83	24.01	24.16					
WC (cm)	**Girls**	62.46	72.26	70.13			*	*ns*	*ns*
(*n* = 58)	(*n* = 23)	(20.64)	(8.86)	(8.36)	*F* (1.103, 61.744) =	.004			

	**Boys**	73.17	80.51	79.83	.230 *(ns)*		***	*ns*	**
	(n = 35)	21.04	15.70	14.44					
**Aerobic Fitness**									
VO_2max	**Girls**	36.96	35.09	36.21			**	*	*ns*
(ml/kg/min)	(n = 38)	(3.40)	(2.78)	(3.25)	F (1.613, 141.922) =	.001			
(n = 90)	**Boys**	46.36	44.77	45.76	.074 *(ns)*		***	***	*ns*
	(n = 52)	6.59	7.36	7.71					
**Neuromuscular Fitness**									
	**Girls**	32.49	30.41	35.35			*ns*	*ns*	*ns*
Sit-ups	(n = 37)	(18.27)	(20.81)	(20.95)	F (1.768, 139.698) =	.010			
(n = 81)	**Boys**	48.09	44.68	46.86	.798 *(ns)*		*ns*	*	*ns*
	(n = 44)	22.52	24.10	23.04					
	**Girls**	7.61	6.81	9.25			*ns*	**	*ns*
Push-ups	(n = 36)	(5.62)	(4.57)	(4.99)	F (1.774, 143.722) = 1.342 *(ns)*	.016			
(n = 83)	**Boys**	15.04	16.09	18.49			*ns*	***	**
	(n = 47)	8.88	9.60	10.26					
	**Girls**	135.97	132.82	133.50			*ns*	*ns*	*ns*
Horizontal Jump	(n = 38)	(19.64)	(19.88)	(19.92)	F (1.803, 155.060) = 3.503*	.039			
(n = 88)	**Boys**	179.54	184.32	187.68			*ns*	*ns*	**

	(*n* = 50)	36.46	34.02	36.02					
20m Run (*n* = 93)	**Girls** (*n* = 41)	4.29 (0.47)	4.28 (0.30)	4.24 (0.34)	*F* (1.405, 127.838) = .702 (*ns*)	.008	*ns*	*ns*	*ns*
	**Boys** (*n* = 52)	3.79 0.63	3.70 0.49	3.70 0.46			*ns*	*ns*	*ns*
Sit and Reach (*n* = 439)	**Girls** (*n* = 41)	26.10 (7.56)	23.62 (7.27)	26.85 (6.59)	*F* (1.866, 162.336) = .356 (*ns*)	.004	*	***	*ns*
	**Boys** (*n* = 48)	18.59 (6.46)	16.71 (8.02)	19.08 (7.30)			*ns*	**	*ns*

*$p < 0.05$, **$p < 0.01$, ***$p < 0.001$; *ns* – non-significant value; BMI HFZc – body mass index health fitness zone continuum; WC – waist circumference; VO2max - maximal oxygen uptake.
